# Incidence and outcomes of Merkel cell carcinoma related to Merkel cell polyomavirus status in Iceland in 1981-2023

**DOI:** 10.1016/j.jdin.2024.06.004

**Published:** 2024-07-14

**Authors:** Maria Vygovska, David Hoyt, Ashley M. Snyder, Thorarinn Jonmundsson, Ashley Khouri, Dev Ram Sahni, Jonathan Ungar, Jesse M. Lewin, Nicholas Gulati, Robert G. Phelps, Vikram N. Sahni, Jane M. Grant-Kels, Helgi Sigurdsson, Jon Gunnlaugur Jonasson, Jonas A. Adalsteinsson

**Affiliations:** a Division of Pathology, Landspitali-University Hospital, Reykjavik, Iceland; b Division of Dermatology, Spencer Fox Eccles School of Medicine, University of Utah, Salt Lake City, Utah; c Department of Dermatology, University of Utah, Salt Lake City, Utah; d Department of Population Health Sciences, University of Utah, Salt Lake City, Utah; e Division of Epidemiology, Department of Internal Medicine, University of Utah, Salt Lake City, Utah; f Faculty of Medicine, Division of Oncology, University of Iceland, Reykjavik, Iceland; g Department of Dermatology, Brigham and Women’s Hospital, Boston, Massachusetts; h Department of Dermatology, Icahn School of Medicine at Mount Sinai, New York, New York; i Department of Dermatopathology, Icahn School of Medicine at Mount Sinai, New York, New York; j Department of Dermatology, University of Connecticut School of Medicine, Farmington, Connecticut; k Department of Dermatology, University of Florida College of Medicine, Gainesville, Florida

**Keywords:** basal cell carcinoma, Iceland, Merkel cell carcinoma, photodamage, polyomavirus, squamous cell carcinoma

## Abstract

**Background:**

Impact of Merkel cell polyomavirus (MCPyV) associated Merkel cell carcinoma (MCC) has not been assessed in the Icelandic population, nor in a whole population elsewhere.

**Objectives:**

The primary objective was to assess trends in the incidence of MCC in Iceland and the association with MCPyV. Secondary objectives aimed to analyze MCC outcomes.

**Methods:**

In this retrospective cohort study, patients diagnosed with MCC between 1981 and 2021 were identified from the Icelandic Cancer Registry. Patients were separated into 2 groups based on MCPyV immunochemistry staining. Age-standardized incidence was calculated and Joinpoint analysis was used to assess incidence trends. A Cox proportional hazards model was used to assess survival differences between the 2 groups.

**Results:**

Overall incidence of MCC increased from 0.015 to 0.26 per 100,000 persons, though the incidence of MCPyV positive cases recently decreased while negative cases increased. MCPyV negative tumors were associated with sun exposure (*P* < .01), a history of keratinocyte carcinoma, smaller tumor size, and lower overall survival.

**Limitations:**

Even with population-level data, comprehensively investigating associations with MCC is difficult due to its rarity.

**Conclusion:**

MCPyV negative MCC tumors were associated with lower survival despite smaller tumor size. Thus, MCPyV status could be an important prognostic biomarker.

**Capsule Summary**

• The impact of Merkel cell polyomavirus associated with Merkel cell carcinoma has not been studied in Iceland.
• Incidence of polyomavirus negative Merkel cell carcinoma is on the rise in Iceland possibly due to increased sun exposure. Negative tumors were associated with history of keratinocyte carcinoma and a more aggressive clinical course.

## Introduction

Merkel cell carcinoma (MCC) is a rare aggressive form of skin cancer arising from uncontrolled growth of cells that share features with Merkel cells, which are mechanoreceptors in the epidermis of the skin.^1^^,^^2^ MCC is most commonly identified in people over 65 years old with fair skin. It typically presents as a solitary, erythematous, or violaceous, rapidly enlarging nodule on the head or extremities.^1^^,^^3^ The 5-year overall survival is 40% according to one large cohort study.^4^ Though there is still debate over whether MCC arises directly from mutant Merkel cells, it has been established that MCC develops via 2 pathogenic mechanisms: viral genomic integration of the Merkel cell polyomavirus (MCPyV) or spontaneous mutation after DNA damage.^5^

MCPyV was first discovered in 2008 and subsequently identified as a contributing factor in 80% of MCCs. It has been theorized that the other 20% of MCCs are caused in large part by ultraviolet (UV) radiation.^6^ The relationship between MCPyV status and outcomes is not well established, with conflicting studies on associations between viral status and overall survival.^7^^,^^8^

Increased incidence of MCC has been of recent concern in countries such as France,^9^ Sweden,^10^ the Netherlands,^11^ England,^12^^,^^13^ Australia,^14^^,^^15^ and the United States.^16^ Whole population data on the relationship between MCC and MCPyV are lacking. Understanding the complex interplay between MCPyV status, sun exposure, and immunosuppression in combination with other factors is important. The incidence of basal cell carcinoma and squamous cell carcinoma is increasing in Iceland, likely due to increased population-wide sun exposure and diagnosis via expanding dermatology access.^17^, ^18^, ^19^ Analyzing the incidence of MCC in this context may yield valuable information as it is less likely to be overdiagnosed due to its aggressive nature, in contrast with the majority of keratinocyte carcinomas. The relationship between MCPyV and MCC incidence may be difficult to analyze since MCPyV status is usually not assessed at the time of diagnosis. Iceland provides a unique opportunity to study the incidence of MCC and its relationship with MCPyV in a whole population. The Icelandic Cancer Registry documents every MCC diagnosed in Iceland. Every case is pathologically confirmed, and every patient has a national identifier that can be used to link to electronic medical records with virtually no loss to follow-up due to nationwide registration.^20^

Considering the increasing global incidence of MCC and lack of nationwide MCPyV staining data, the purpose of this study was to explore:

1. The incidence of MCC in a whole population which is exposed to relatively low levels of UV radiation.
2. The impact of MCPyV positive versus negative tumors on this incidence, and connection to previous sun exposure.
3. The differences in survival of patients diagnosed with MCPyV positive and negative tumors.

## Materials and methods

In this retrospective cohort study, all patients diagnosed with MCC between the years 1981 and 2021 were identified from the Icelandic Cancer Registry. Pathology specimens were retrieved, stained for MCPyV using CM2B4 (MCPyV large T-antigen), and reviewed by a pathologist for diagnostic confirmation. Any degree of nuclear staining in tumor cells was considered positive. This study was approved by the National Bioethics Committee of Iceland (VSN, 18-071).

### Demographics

Demographic information, including age at diagnosis (years) and sex, was recorded. Clinical characteristics included MCC stage at diagnosis, location of primary tumor, tumor size in millimeters (mm), survival after diagnosis (months), treatment with chemotherapy or radiation therapy, history of other skin cancers, and MCPyV tumor status. History of prior sun exposure was evaluated at the time of diagnosis—by patient questionnaire and clinical assessment—and recorded as “high” or “low.” A patient was classified as “high” if clinically visible and diffuse photodamage was present at the time of diagnosis (including multiple solar lentigines, poikiloderma, and solar elastosis, with or without the presence of actinic keratoses). In order to further stratify patients with a significant history of UV exposure, a history of keratinocyte carcinoma (either squamous cell carcinoma or basal cell carcinoma) was also documented as a surrogate risk factor.

### Statistical analyses

Patients were separated into 2 groups based on MCPyV stain results. Fisher’s exact test was utilized to assess the association between MCPyV status and demographic and clinical categorical variables. Medians were assessed using the Wilcoxon rank-sum test. Age-specific rates were calculated using Icelandic census data. Rates were age-standardized using the Segi-Doll world standard population^21^ and Icelandic population data. The incidence was then stratified by MCPyV status. Ten-year running averages of MCC rates between 1981 and 2021 were calculated. Running averages were deemed appropriate to better assess changes over time due to the rarity of MCC. Joinpoint analysis was performed to identify significant changes in incidence trends.

Kaplan-Meier estimation was used to examine overall and MCC-specific survival in the cohort. Observed differences in survival based on MCPyV stain results were tested with the log-rank test. Univariate Cox proportional hazards models were fitted to quantify the effect of age, stain result, sex, tumor size, and stage at diagnosis on survival. Multivariate regression models were not considered due to the small sample size. Proportional hazards assumptions were examined using log-log plots and Schoenfield residuals.

An α < 0.05 was considered statistically significant for two-sided tests. SAS, version 9.4 (SAS, Cary), Stata, version 16.1 (StataCorp), R, version 4.3.2, and Joinpoint, version 5.0.2 software were used for all statistical analyses.

## Results

Cohort characteristics are summarized in Table I. Thirty-four patients with MCC were identified in this Icelandic cohort; 33 (97.1%) had an MCPyV stain recorded as pathology samples from one tumor were unobtainable. Among these 33 patients, there were 17 deaths observed, of which 10 were MCC specific. Patients with MCPyV negative tumors more often had a history of nonmelanoma skin cancer (*P* = .04) and more frequently were determined to have clinically significant photodamage on exam (*P* < .01). The median age between groups when stratified by MCPyV status was 77 for positive and 78 for negative tumors. There were slightly more female patients overall (54.5%) and in each stain status group. Two patients were transplant recipients (one kidney and one bone marrow), and both were in the MCPyV negative group.

**Table I:** Merkel cell carcinoma demographics and select clinical and lifestyle characteristics by Merkel cell polyomavirus status

Variable	Total	Negative	Positive	*P* value
Demographics				
*N*	33∗	13 (39.4%)	20 (60.6%)	
Age at dx (years)	77 [72-84]	78 [75-83]	77 [71-84]	.59
Women	18 (54.5%)	7 (53.8%)	11 (55.0%)	1
Survival				
Follow-up (months)	51 [12-88]	19 [9-51]	64 [14-119]	.06
Any death	17 (51.5%)	7 (53.8%)	10 (50.0%)	1
MCC-specific death	10 (30.3%)	7 (53.8%)	3 (15.0%)	.03
Tumor characteristics				
Stage at dx				.14
I	12 (36.4%)	4 (30.8%)	8 (40.0%)	
II	12 (36.4%)	3 (23.1%)	9 (45.0%)	
III	1 (3%)	1 (7.7%)	0 (0.0%)	
IV	7 (22.2%)	5 (38.5%)	2 (10.0%)	
Missing	1 (3.0%)	0 (0.00%)	1 (5.0%)	
Tumor location				<.01
Upper limb	9 (27.3%)	1 (7.7%)	8 (40%)	
Lower limb	3 (9.1%)	1 (7.7%)	2 (10%)	
Trunk	8 (24.2%)	8 (61.5%)	0 (0.0%)	
Head/neck	12 (36.4%)	3 (23.1%)	9 (45%)	.5
Tumor size† (mm)	20 [15-35]	18 [14-34]	20 [15-36]	
Risk factors				
High level of sun exposure	11 (33.3%)	8 (61.5%)	3 (15%)	<.01
History of non-melanoma skin cancer	15 (45.5%)	9 (69.2%)	6 (30%)	.04
Treatment				
Any therapy	15 (45.5%)	9 (69.2%)	6 (30%)	.04
Chemotherapy	3 (9.1%)	3 (23.1%)	0 (0%)	.05
Radiation therapy	15 (45.5%)	9 (69.2%)	6 (31.6%)	.04

Median (interquartile range) for continuous variables and *n* (%) for categorical variables.

*dx*, Diagnosis; *MCC*, Merkel cell carcinoma; *mm*, millimeters.

∗ *N* = 33, missing value *n* = 1 for patient who had unobtainable pathology.

† *N* = 32; missing value of *n* = 1 not included in calculating *P* value.

### Incidence

**Fig 1:**
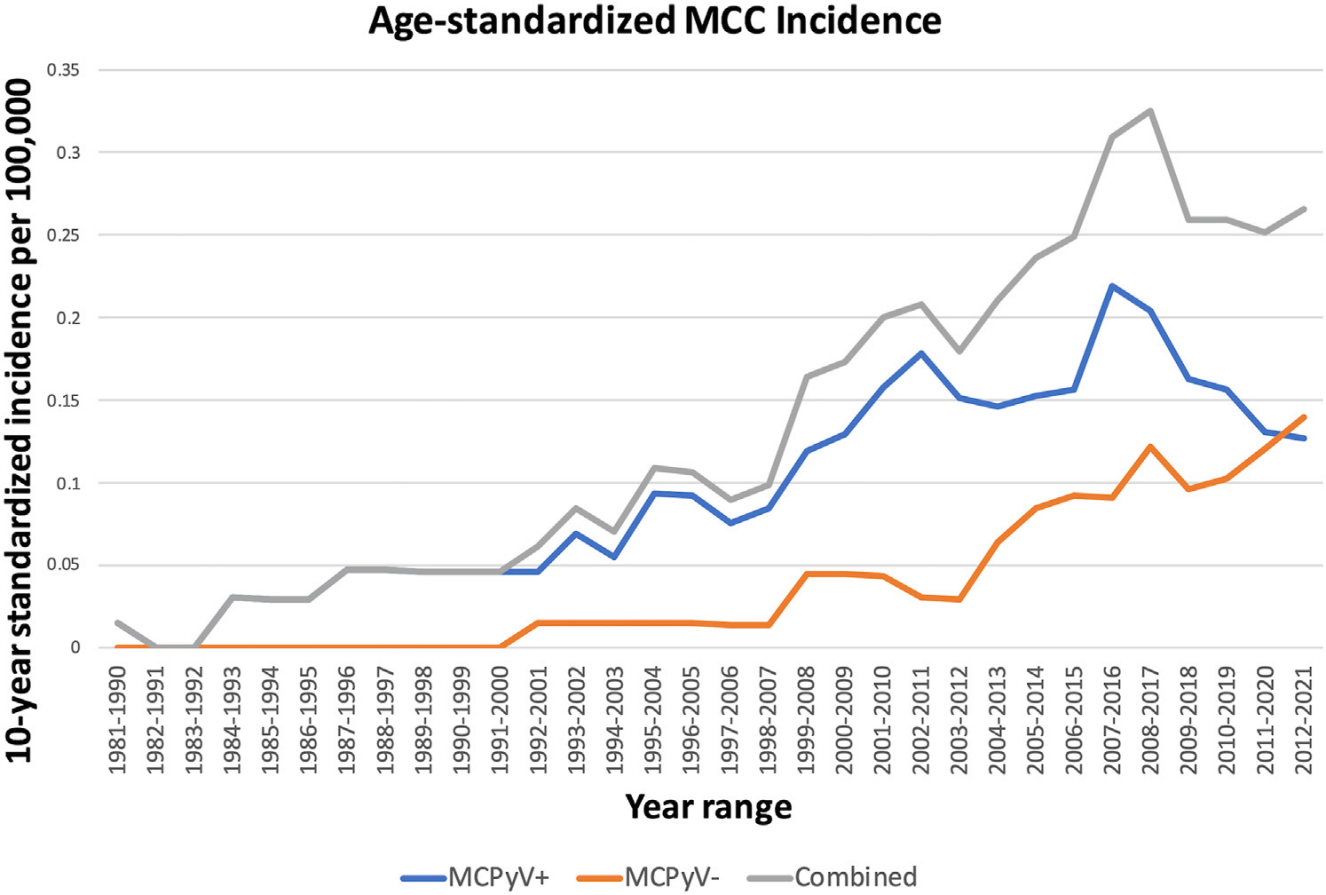
Age-standardized incidence of Merkel cell carcinoma (MCC) in Iceland per 100,000 person-years, looking at total MCC counts (*gray*), Merkel cell polyomavirus (MCPyV) positive tumors (*blue*) and MCPyV negative tumors (*orange*) (10-year running averages).

**Fig 2:**
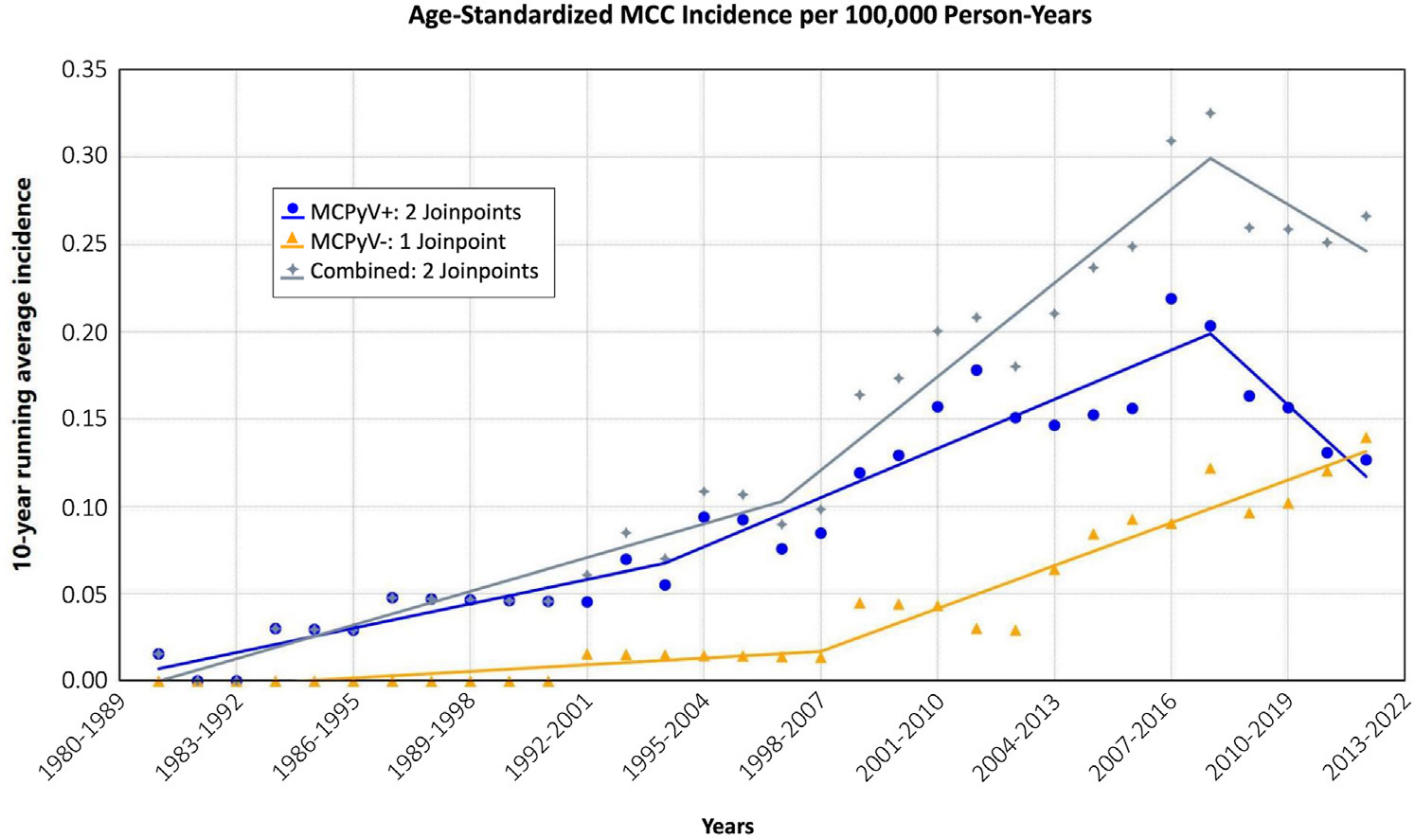
Joinpoint analysis evaluating slope and annual percent change in age-standardized incidence per 100,000 person-years of Merkel cell polyomavirus (MCPyV) positive (*blue*), MCPyV negative (*orange*), and total Merkel cell carcinoma counts (*gray*).

While some fluctuation occurred in age-standardized incidence throughout the study period, overall incidence increased between 1981 and 2021. The incidence of all MCC tumors increased from 0.015 to 0.26 per 100,000 people over the duration of the study. The incidence of MCPyV positive tumors began to decline in the 2007-2016 time period, while the incidence of MCPyV negative tumors continued to rise (Fig 1). Joinpoint analysis confirmed a change in incidence which resulted in a downward trend in overall MCC and MCPyV positive tumors between 2008 and 2021 (Fig 2). Incidence of total cases of MCC increased more sharply starting in the late 1990s. MCPyV positive tumors were rare in the 1980s and 1990s, with only 3 cases diagnosed during that time period; there were no MCPyV negative tumors during these decades (Figs 1 and 2).

### Survival

**Fig 3:**
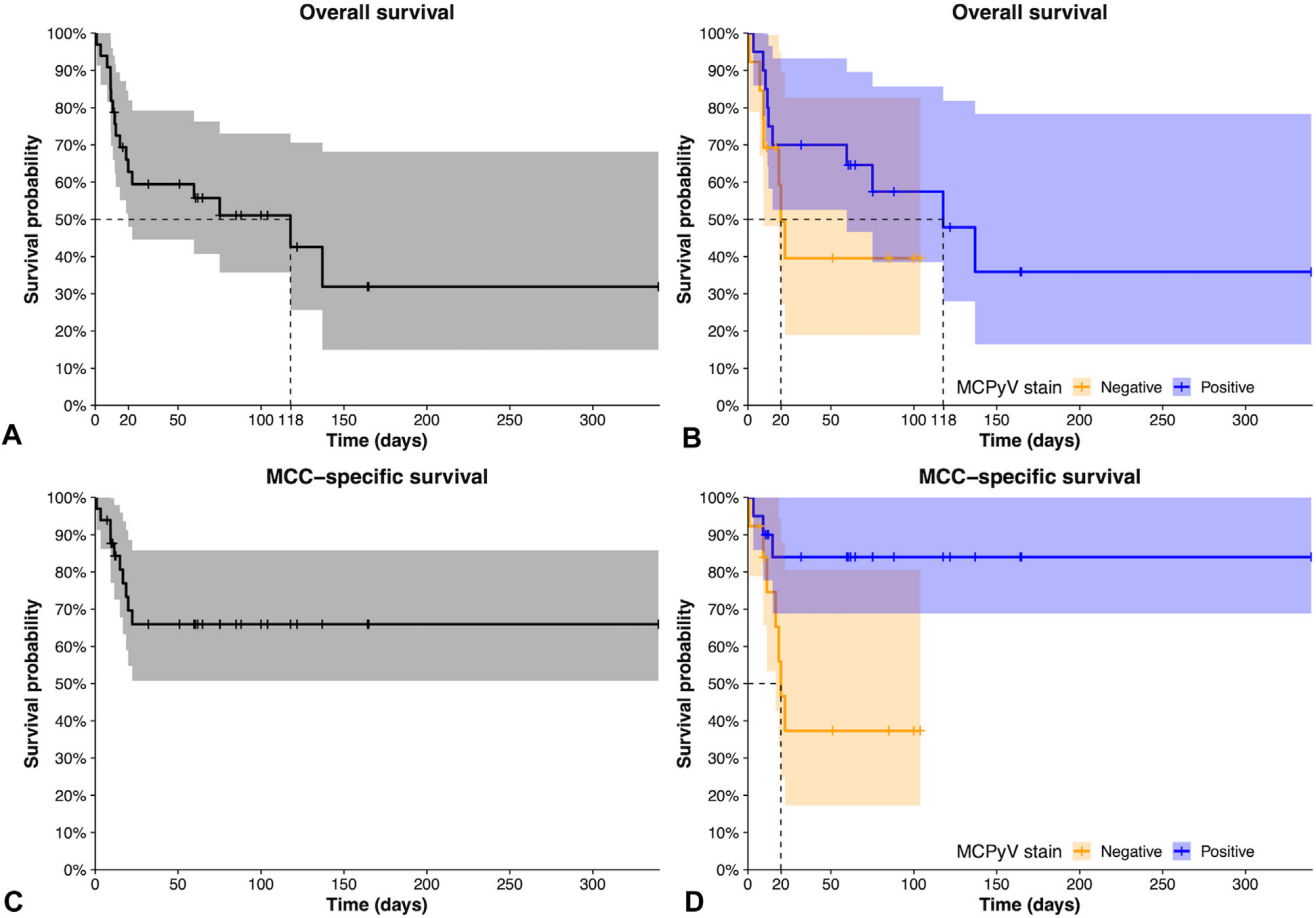
Kaplan-Meier estimation of overall (*above*) and Merkel cell carcinoma (MCC)-specific (*below*) survival in the cohort (*N* = 33). Survival was stratified by Merkel cell polyomavirus (MCPyV) (*yellow* and *blue*). Included are 95% confidence intervals and median survival (depicted by *dotted lines*). **A,** Overall survival, not stratified by MCPyV status. **B,** Overall survival, stratified by MCPyV status. **C,** MCC-specific survival, not stratified by MCPyV status. **D,** MCC-specific survival, stratified by MCPyV status.

Patients with MCPyV positive tumors experienced a median follow-up that was more than triple that of the MCPyV negative group (64 vs 19 months, *P* = .06; Table I). The Cox regression is summarized in Supplementary Table I, available via Mendeley at https://data.mendeley.com/datasets/t3mtsb6rp6/1. The survival experience of the cohort is illustrated with Kaplan-Meier plots (Fig 3) for both overall and MCC-specific survival. The survival of MCPyV positive patients was observed to be greater; a log-rank test showed a significant difference in survival (*P* = .02) when MCC-specific death was considered.

Cox proportional hazards regression was used to further assess associations between MCPyV stain results and MCC-specific death. MCPyV positive status was protective (HR [95% CI] = 0.22 [0.06-0.86]) and significant (*P* = .03). Age (1.04 [0.97-1.12], *P* = .22) and tumor size (1.00 [0.97-1.04], *P* = .75) effects were around the null. Comparing stages III and IV cancer to stages I and II as the reference produced a large effect size, though not statistically significant, perhaps due to the small sample size (*N* = 31, 3.00 [0.86-10.3], *P* = .08). The log-log and Schoenfeld plots did not suggest a departure from the proportional hazard assumption (Supplementary Figs 1 and 2, available via Mendeley at https://data.mendeley.com/datasets/t3mtsb6rp6/1).

## Discussion

Population-level data from the Icelandic Cancer Registry were used to understand the incidence of MCC over time in Iceland and the relationship between MCPyV and MCC. Because data were obtained from a population-level registry, there should be minimal loss to follow-up. We discovered that the incidence of MCC is increasing, driven by a recent uptick in the incidence of MCPyV negative tumors, which were associated with a previous history of keratinocyte carcinoma and sun exposure in most cases. This is concerning as evidence suggests that this might be the more aggressive variant of the 2.^22^ While this study was not designed to assess the prognostic value of MCPyV status in a prospective manner, our results indicate that the absence of MCPyV might portend a worse outcome. Since MCPyV negative tumors are influenced by sun exposure,^23^^,^^24^ our results could be generalizable to other light-skinned populations. This finding is concordant with other light-skinned populations living in high UV areas, such as in the United States, which is also experiencing a rise in incidence of MCC.^16^

### Incidence

Iceland is home to the world's lowest average UV-index capital city, making it a particularly informative setting to study isolated risk factors in skin cancers.^25^ As with many other countries,^9^, ^10^, ^11^, ^12^, ^13^, ^14^, ^15^ including the United States,^16^ MCC incidence in Iceland has increased over the past 40 years. In our study, this increase was associated with a rise in MCPyV negative tumors, while MCPyV positive tumor rates began to decrease. A recent study demonstrated virus negative tumors had a stronger association with disease progression and only represented about 20% of all MCCs.^22^

Given the lower survival in patients with MCPyV negative tumors in this population, it is important to identify factors that may be driving the increased incidence of MCPyV negative MCCs. MCPyV positive tumors primarily occurred on the head/neck and upper limbs but did not occur on the trunk. With over 50% of MCPyV negative MCCs occurring on the trunk in our cohort, exposure to tanning beds and the increased popularity of sunbathing may be relevant factors for this otherwise less exposed region of the body. Tanning bed usage increased sharply from 1979 to 2004 in Iceland and is still higher than in neighboring countries despite a decline in use since 2004.^26^ Another possible factor is foreign travel, which increased more than ten-fold between 1970 and 2006 among Icelanders and could be a source of further UV exposure.^27^ Previous studies have indicated that UV radiation could help regulate cellular processes related to MCPyV genes,^28^^,^^29^ so it is possible that UV radiation combined with MCPyV may trigger MCC. MCC develops through 2 distinct routes of carcinogenesis: gene transformation of MCPyV-encoded genes and spontaneous mutation of UV-damaged DNA associated with increased age, exposure, and weakened immunity.^7^ Though MCPyV positivity remains a clinically relevant risk factor, it is not fully understood how a virus found in 49% to 80% of lesion-free skin samples undergoes neoplastic differentiation in a small fraction of patients, nor how to prevent that transformation.^30^

Joinpoint analysis demonstrated that a significant increase in the incidence of MCC occurred in a similar time period as when increases in incidence of basal cell carcinoma,^18^ squamous cell carcinoma,^19^ and melanoma^27^ were observed in Iceland. The incidence increase in these other tumors was attributed to a combination of increased sun exposure of the Icelandic population and improved dermatology access. However, due to the aggressiveness of MCC, increased access to dermatology resources and subsequent improvements in diagnosing MCC cannot explain the incidence increase seen in this study. This further supports the hypothesis that while increased screening might play some role in the diagnosis of other types of skin cancers seen in Iceland, a true increase in population-wide sun exposure is likely occurring and playing a significant role. Our results confirmed that the majority of MCPyV negative cases were both associated with a prior history of either squamous cell or basal cell carcinoma, and a significant prior history of sun exposure.

Joinpoint analysis identified a recent downtrend in overall MCC incidence (due to a decrease in MCC positive tumors). A similar trend has been observed with invasive melanoma in Iceland in recent years, which was attributed to population-wide awareness and decreased tanning bed use in recent years.^27^ However, the increase in MCPyV negative tumors observed in this study contradicts this.

### MCPyV status and outcomes

Since its discovery in 2008, MCPyV has been the focus of many studies evaluating its impact on overall survival in patients with MCC. Several studies have revealed a better prognosis and increased survival in patients with MCPyV positive tumors, though several others have shown the inverse or no association at all.^1^^,^^7^^,^^31^, ^32^, ^33^, ^34^ In our group of patients, the findings were consistent with those reported in the majority of literature, demonstrating better overall survival in patients with MCPyV positive tumors. Although the proportional hazards assumption was not rejected, the results must be interpreted carefully due to the small sample size. In the Cox analysis (Supplementary Table I, available via Mendeley at https://data.mendeley.com/datasets/t3mtsb6rp6/1), stage at diagnosis and stain type become more extreme when MCC-specific death is considered, with hazard ratios being in line with the literature.^1^^,^^7^ This could be due to many reasons. A higher number of patients in the MCPyV negative group presented with stage IV disease (38.5% vs 10.0%), which subsequently increased the likelihood that this group of patients received both chemotherapy and radiation therapy. In this context, while not significant, tumor size was lower in the MCPyV negative group (18 vs 20 mm). Despite this smaller tumor size on presentation, more people presented with stage IV disease at the time of diagnosis, suggesting that MCPyV status might be a more important factor than tumor size when it comes to prognostic value, although our study was not powered to assess this relationship. Additionally, the 2 transplant patients who developed MCC in our study had MCPyV negative tumors. We expected the opposite to be the case, as virus reactivation in the setting of transplant-related immunosuppression could lead to MCPyV positive MCC.^35^

### Limitations

With a reported incidence of 0.1-0.3 cases yearly per 100,000 people in Nordic countries, the rarity of this cancer makes it difficult to study.^10^^,^^36^ Subsequently, it is challenging to isolate and analyze individual risk factors. Future analyses could benefit from compiling MCC data from several countries to generate a larger sample size of MCC patients. A model that can account for confounders such as age and pre-existing comorbidities can better isolate the effect of MCPyV status. However, this could also pose a limitation since populations from other countries may have different common risk factors that could influence incidence over time. While the CM2B4 stain is highly sensitive and specific for MCC (up to 94% and 100% respectively), it is still inferior to PCR which we were unable to perform.^22^^,^^37^ Finally, our population-wide data potentially limits the generalizability of our results.

## Conclusion

This whole population study examining MCC and the impact of MCPyV demonstrated a continued increase in the incidence of MCPyV negative tumors, a potentially aggressive subset associated with sun exposure. These tumors more often presented with stage IV disease, despite their smaller size. This suggests that MCPyV status may be an important prognostic factor for MCC, although this needs further validation in future studies.

## Conflicts of interest

None disclosed.
